# Characterization of hearing-impairment in Generalized Arterial Calcification of Infancy (GACI)

**DOI:** 10.1186/s13023-022-02410-w

**Published:** 2022-07-19

**Authors:** Elizabeth H. Theng, Carmen C. Brewer, Ralf Oheim, Christopher K. Zalewski, Kelly A. King, Maximillian M. Delsmann, Tim Rolvien, Rachel I. Gafni, Demetrios T. Braddock, H. Jeffrey Kim, Carlos R. Ferreira

**Affiliations:** 1 Skeletal Disorders and Mineral Homeostasis Section, National Institute of Dental and Craniofacial Research, National Institutes of Health, Bethesda, MD USA; 2 Audiology Unit, Otolaryngology Branch, National Institute on Deafness and Other Communication Disorders, National Institutes of Health, Bethesda, MD USA; 3 Martin Seitz Center for Rare Diseases, University Medical Center Hamburg-Eppendorf, Hamburg, Germany; 4 Department of Trauma and Orthopaedic Surgery, Division of Orthopaedics, University Medical Center Hamburg-Eppendorf, Hamburg, Germany; 5 Department of Pathology, Yale University School of Medicine, New Haven, CT USA; 6 Office of Clinical Director, National Institute on Deafness and Other Communication Disorders, National Institutes of Health, Bethesda, MD USA; 7 Department of Otolaryngology–Head & Neck Surgery, District of Columbia, Georgetown University Hospital, Washington, USA; 8 Medical Genomics and Metabolic Genetics Branch, National Human Genome Research Institute, National Institutes of Health, 49 Convent Dr, Building 49, Room 4A38, Bethesda, MD 20892 USA

## Abstract

**Background and importance:**

Hearing loss (HL) has been sporadically described, but not well characterized, in Generalized Arterial Calcification of Infancy (GACI), a rare disease in which pathological calcification typically presents in infancy.

**Objectives:**

This study aims to describe the clinical audiologic and otologic features and potential etiology of hearing impairment in GACI and gain pathophysiological insight from a murine model of GACI.

**Design:**

Cross-sectional cohort study of individuals with GACI. Murine ossicle micromorphology of the *ENPP1*^*asj/asj*^ mutant compared to wild-type.

**Setting:**

Clinical research hospital; basic science laboratory.

**Participants:**

Nineteen individuals with GACI who met clinical, biochemical, and genetic criteria for diagnosis.

**Main outcomes and measures:**

Clinical, biochemical, and radiologic features associated with hearing status.

**Results:**

Pure-tone thresholds could be established in 15 (n = 30 ears) of the 19 patients who underwent audiological assessments. The prevalence of HL was 50% (15/30) of ears, with conductive HL in 80% and sensorineural HL in 20%. In terms of patients with HL (n = 8), seven patients had bilateral HL and one patient had unilateral HL. Degree of HL was mild to moderate for 87% of the 15 ears with hearing loss. Of those patients with sufficient pure-tone and middle ear function data, 80% (8/10) had audiometric configurations suggestive of ossicular chain dysfunction (OCD). Recurrent episodes of otitis media (ROM) requiring pressure-equalizing tube placement were common. In patients who underwent cranial CT, 54.5% (6/11) had auricular calcification. Quantitative backscattered electron imaging (qBEI) of murine ossicles supports an OCD component of auditory dysfunction in GACI, suggesting loss of ossicular osteocytes without initiation of bone remodeling.

**Conclusions and relevance:**

Hearing loss is common in GACI; it is most often conductive, and mild to moderate in severity. The etiology of HL is likely multifactorial, involving dysfunction of the ossicular chain and/or recurrent otitis media. Clinically, this study highlights the importance of early audiologic and otologic evaluation in persons with GACI. Novel findings of high rates of OCD and ROM may inform management, and in cases of unclear HL etiology, dedicated temporal bone imaging should be considered.

**Supplementary Information:**

The online version contains supplementary material available at 10.1186/s13023-022-02410-w.

## Background

Generalized Arterial Calcification of Infancy (GACI) is a rare pediatric condition characterized by arterial calcification in early infancy, hypophosphatemic rickets, pseudoxanthoma elasticum, and hearing loss (HL) [1, 2]. To date, only ~ 200 cases of GACI have been reported, with an estimated prevalence of 1 in 200,000 [2, 3]. Most GACI cases are due to biallelic variants in the gene encoding ectonucleotide pyrophosphatase/phosphodiesterase-1 (*ENPP1*) leading to ENPP1 enzyme deficiency, and less commonly due to variants in ATP-binding cassette sub-family C member 6 (*ABCC6*) [4]. ENPP1 is a transmembrane glycoprotein that cleaves extracellular adenosine triphosphate (ATP) into adenosine monophosphate (AMP) and pyrophosphate (PP_i_), the latter of which is a potent inhibitor of mineralization. Although the exact molecule is unknown, ABCC6 is thought to transport ATP into the extracellular space, providing substrate for ENPP1 and being indirectly responsible for PP_i_ production. Clinically, variable cardiovascular findings predominate in infancy, including widespread arterial calcification, heart failure, respiratory distress, cyanosis, edema, hypertension, and/or cardiomegaly. In survivors, mineral dysregulation and skeletal complications arise, manifesting as hypophosphatemic rickets, peri-articular and entheses calcifications, and cervical spine fusion [1, 2, 4, 5]. In addition, typical skin and retinal features of pseudoxanthoma elasticum (PXE) and HL have been noted [1, 6, 7].

Reports of hearing status in GACI have been limited in scope; case reports and retrospective reviews have described conductive, sensorineural, and mixed HL diagnosed in infancy or early childhood [2, 4, 7, 8]. There has only been one murine study evaluating auditory dysfunction in ENPP1 deficiency, in which the *ENPP1*^*asj/asj*^ mouse, a model of ENPP1 deficiency, developed progressive conductive HL (CHL), otitis media with effusion, and features of ectopic mineralization of the middle ear structures, including fusion of the malleus and incus and a thickened and overly calcified stapedial artery [9]. Given the rarity of disease, auditory function has not been systematically explored in a cohort of patients with GACI. This study aims to describe the extent, severity, complications, and potential etiology of HL in affected individuals.

## Methods

Twenty patients with GACI confirmed by *ENPP1* or *ABCC6* pathogenic variants were referred for audiologic and otologic evaluations from 2013 to 2020 at the National Institutes of Health (NIH) Clinical Center, Bethesda, Maryland, USA. Patients were referred from patient advocacy groups and physicians, and all were enrolled in research protocols NCT00369421 and NCT00024804. These studies were conducted in accordance with the Declaration of Helsinki and protocols were approved by the NIH Institutional Review Board (IRB). All subjects or their guardians provided informed consent and assent when appropriate.

Standard audiometric measures included, when possible, air- and bone-conduction pure-tone thresholds for 250 to 8000 Hz and 250 to 4000 Hz, respectively. Clinically significant HL was defined as a 4-frequency (0.5/1/2/4 kHz) pure-tone average (4F-PTA) > 20 decibels hearing level (dBHL). Degree of HL was categorized as mild, moderate, severe, or profound [10] (Additional file 1: Table S1). Type of HL was determined using a 3-frequency (0.5/1/2 kHz) pure-tone average (3F-PTA) and was classified as CHL, sensorineural (SNHL), mixed (MHL), or as having a subclinical conductive component (Additional file 1: Table S1). For patients with serial tests, the most recent audiometric evaluation was used for analysis.

Tympanometry and acoustic stapedial reflex thresholds (0.5/1/2 kHz) were conducted to evaluate middle ear function and distortion product otoacoustic emissions (DPOAE) were evaluated to assess cochlear function. Details and interpretation of these procedures are presented in the supplemental materials (Additional file 1: Table S1).

**Fig. 1:**
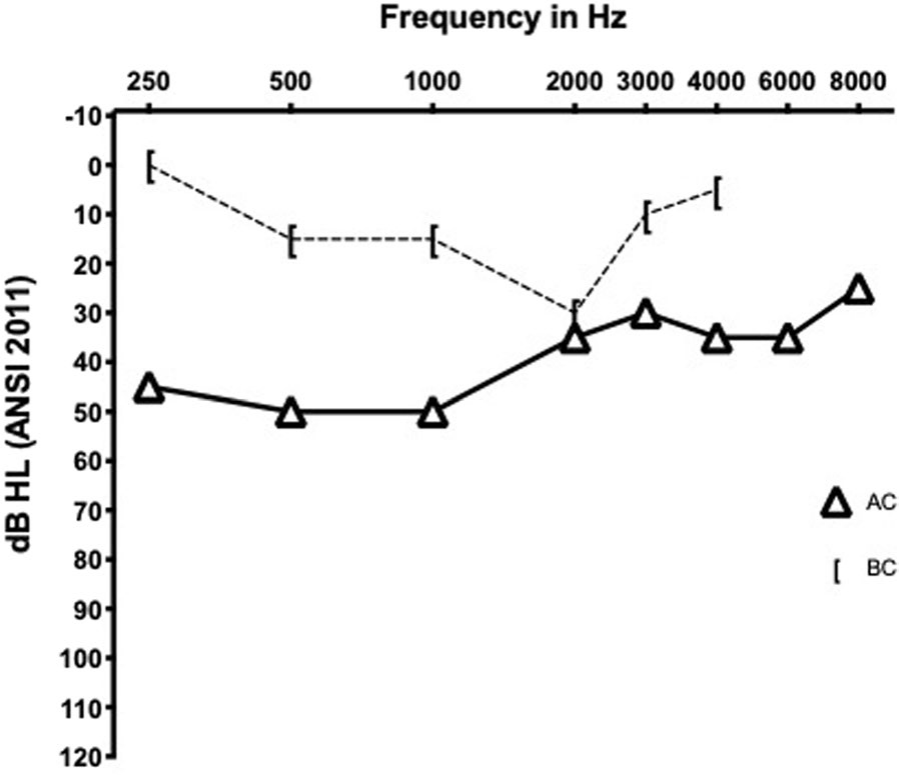
Carhart notch example. Pure-tone audiogram from the right ear of a 25-year-old man with GACI showing a conductive hearing loss with narrowing of the air–bone gap at 2000 Hz characteristic of a Carhart notch. This patient’s tympanogram is normal (Type A) and acoustic stapedial reflexes are absent

Ossicular chain dysfunction (OCD) was defined as: normal hearing and normal tympanogram with elevated or absent acoustic reflexes, or normal tympanogram with air-bone gaps of > 10 dB, or presence of a Carhart-notch (e.g., Fig. 1). The Carhart-notch is a characteristic artifactual worsening in bone conduction threshold and narrowing of the air–bone gap, typically most pronounced at 2 kHz and suggestive of ossicular chain fixation [11].

Computed tomography (CT) of the head/skull was performed, and in selective cases, temporal bone CT (TBCT) with section cuts of 0.6 mm were evaluated for morphologic abnormalities in the middle ear and otic capsule.

For one patient with bilateral enlarged vestibular aqueducts, an imaging finding that was unusual in this setting, research exome sequencing was pursued to rule out a second etiology. DNA was extracted from peripheral blood mononuclear cell and samples were prepared according to an Agilent SureSelect Target Enrichment Kit preparation guide. The libraries were sequenced with Illumina HiSeq 2000/2500 sequencer. Alignment to the hg19 reference was performed with the Burrows–Wheeler aligner (BWA) [12], deduplication with Picard, and GATK best practice guidelines were followed for variant analysis [13]. Annotation was performed via SnpEff [14].

Since most GACI survivors develop hypophosphatemic rickets and vascular disease over their lifetimes, we evaluated biochemical markers, factors related to mineral homeostasis, and measures of cardiovascular burden of disease. These included intact and C-terminal FGF23 (a phosphate-regulating hormone), blood phosphate, calcitriol and phosphorus supplementation, age of onset of vascular calcifications, the number of ongoing/resolved antihypertensive/heart failure medications, and bisphosphonate use. C‐terminal FGF23 was measured at Mayo Clinic Laboratories using human FGF23 C-terminal ELISA kits (Immutopics, Quidel, San Diego, CA, USA). Intact FGF23 levels were measured in-house from plasma using human FGF23 intact ELISA kits (Immutopics, Quidel, San Diego, CA, USA).

### Murine model

To characterize mineralization changes that may account for HL, quantitative backscattered electron imaging (qBEI) of the ossicles from *Enpp1*^*asj/asj*^ mutant and wild-type (WT) mice (C57BL6) was performed using standard procedures as described in the methods section of the supplemental materials. The *Enpp1*^*asj/asj*^ mutant [c. 737T > A (p.V246D), initially reported as c.771T > A] is a commercially available murine model of ENPP1 deficiency developed by the Jackson Lab [15]. ‘Asj’ refers to the phenotype of the mutant as it “ages with stiffened joints.”

### Statistical analysis

Descriptive and inferential statistical tests were completed using GraphPad Prism 8 for Windows, version 8.4.1 (GraphPad Software Inc). For comparative analyses, significance for *p*-values was set at < 0.05 and two-tailed, if applicable. Fisher’s exact test was used to compare tympanic membrane findings on otoscopy with hearing status. Spearman correlation was used to evaluate intact and C-terminal FGF23, age of calcification onset, age of bisphosphonate (BP) initiation, duration of BP use, age of rickets treatment initiation, and number of hypertensive/heart failure medications—all individually—with the average of air conduction binaural 4F-PTAs.

## Results

### Prevalence and characteristics of hearing loss in GACI

Twenty patients with GACI due to biallelic pathogenic variants in *ENPP1* or *ABCC6* were referred for audiology testing; one was excluded from analysis due to cerumen impaction (Tables 1 and 2). Pure-tone thresholds were reliably established in 15 patients (10 female, 5 male) with a median age of 8.2 years (range 4.1–38.5), for a total of 30 evaluable ears. In the remaining 4 patients, behavioral audiological evaluations were attempted but unsuccessful due to young age or limited abilities. In these patients, middle ear function and DPOAEs were used to predict peripheral hearing status. Ten of these 19 patients were previously reported [2] and have been marked in Table 2 with an arrow notation. The specific molecular variants found in each of these patients are presented in Additional file 1: Table S2.

**Table 1:** Patient characteristics

	GACI patients time of audiology evaluation (n = 19)	GACI patients with pure-tone data (n = 15)	All patients with hearing loss (n = 8)	Patients with CHL (n = 7*)	Patients with SNHL (n = 2*)
Median age in years (range)	7.9 (0.7–38.5)	8.2 (4.1–38.5)	8.4 (4.1–35.7)	7.9 (4.1–25.5)	22.3 (8.9–35.7)
*Age groups (n)*					
Children (< 10 yrs)	75% (14)	66.7% (10)	75% (6)	83.3% (6*)	50% (1*)
Adolescents (10–19 yrs)	5% (1)	6.7% (1)			
Adults (>/= 20 yrs)	20% (4)	26.7% (4)	25% (2)	16.7% (1)	50% (1)
*Sex*					
Female	60% (11)	66.7% (10)	50% (4)	33.3% (3*)	100% (2*)
Male	40% (8)	33.3% (5)	50% (4)	66.7% (4)	
*Race/ethnicity*					
Black/African American	10.5% (2)	6.7% (1)			
Multiracial	5.3% (1)	6.7% (1)	12.5% (1)	16.7% (1)	
White	84.2% (16)	86.7% (13)	87.5% (7)	83.3% (5)	100% (2)

*CHL* conductive hearing loss, *SNHL* sensorineural hearing loss, *yrs* years

*1 patient with asymmetric bilateral hearing loss is counted as both SNHL (right ear) and CHL (left ear)

**Table 2:** Summary of GACI patients with audiology evaluations (n = 19)

Age (years)	Sex	Right ear					Left ear					Carhart notch	OCD Pattern	History of PET	NBHS Result
		Degree of HL	Type of HL	Tymp	AR	Tympanic Membrane	Degree of HL	Type of HL	Tymp	AR	Tympanic Membrane				
0.7	M			A		Normal			As		Normal			No	Pass
2.0	F			A		Normal			A		Normal			No	Pass
➢4.1	F	Moderate	CHL	A		Normal	Moderate	CHL	A		Normal	No	Yes	No	Fail
➢4.2	M			B (PET)		PET			B (perf)		Thin; PET Extruded			PET	Pass
4.5	F	NH		A		Normal	NH		A		Normal		No	No	Pass
➢5.1	M	NH	NA	C		Minimal fluid under TM	Mild	CHL	B (MEE)		Yellow MEE	No	No	No	Pass
5.8	F	NH	NA	A			NH	NA	A		Normal		No	No	Pass
➢6.3	F	NH		C	Abs		NH		C	Abs	Normal		No		
➢7.0†	M	Moderate	CHL*	A		Thin inferiorly, retracted onto promontory	Severe	CHL*	Ad		Monomeric		Yes	PET	Pass
➢7.9†	F	Mild	CHL*	A	Abs	Normal	Moderate	CHL*	Ad	Abs	Normal	BE	Yes	PET	Pass
➢8.2	M	NH	SubCl	A		Normal	NH	NA	A	Abs	Normal	No	Yes	No	Pass
➢8.9	F	Mild	SNHL	C		SOM	Mild	CHL	C		Retracted, SOM partially filling	RE	Yes	No	Fail
9.0	M			A		Normal			A				No	No	NR
9.7†	M	Moderate	CHL*	A	Abs	Normal	Moderate	CHL*	A	Abs	Thin posteriorly	No	Yes	PET	Fail
13.1	F	NH	NA	A	Elev	Normal	NH	NA	A	Abs	Normal	No	No	No	NR
➢25.5†	M	Moderate	CHL	A	Abs	Thickened, no SOM	Mild	CHL*	A	Abs	Thin, retracted	RE	Yes	PET	NR
26.3	F	NH		A	Abs	Normal	NH	NH	A	Pres	Normal	No	No	No	NR
35.7†^α^	F	Profound	SNHL*	A	Abs	Normal	Profound	SNHL*	Ad	Abs	Normal			PET	Fail
38.5†	F	NH	NA	A	Elev	Normal	NH	NA	A	Elev	Normal	RE	Yes	No	NR

*Abs* absent, *AR* acoustic reflexes, *BE* both ears, *CHL* conductive hearing loss, *Elev* elevated, *HL* hearing loss, *LE* left ear, *MEE* middle ear effusion, *NA* not applicable, *NBHS* newborn hearing screening, *NH* normal hearing, *NR* not reported, *OCD* ossicular chain dysfunction, *PET* past or current pressure equalization tube, *RE* right ear, *SNHL* sensorineural hearing loss, *SOM* serous otitis media, *SubCl* subclinical air-bone gaps, *TM* tympanic membrane (as observed by otoscopy), *Tymp* tympanogram type

➢ Arrows denote nine of ten patients previously reported in Ferreira et al. [2]. The 10th previously reported patient was removed from this study due to cerumen impaction

^†^Daggers denote hearing aid use in the patient

*Asterisks denote hearing aid use in the specific ear

^α^This 35.7-year-old female patient has bilateral EVA and biallelic pathogenic variants in *SLC26A4*

Periods represent insufficient data

Tympanogram types are defined in Additional file 1: Table S1

Eight of the fifteen patients who completed pure-tone testing had HL (Table 2); one had unilateral and seven had bilateral HL, totaling 15 of 30 ears (50%) with HL. Among patients evaluated, 4/10 female patients (40%) and 4/5 male patients (80%) had HL. The median age of the patients with HL was 8.4 years (4.1–35.7).

For ears with HL, the type was conductive in 80% (12/15 ears) and sensorineural in 20% (3/15 ears). A subclinical conductive component was present in a single ear. There were no patients with MHL. Degree of CHL was mild in 33.3% (4/12), moderate in 58.3% (7/12), and severe in 8.3% (1/12). SNHL was mild in one ear (1/3) and bilaterally profound (2/3) in one patient. This patient with profound SNHL had a diagnosis of enlarged vestibular aqueducts (EVA) and biallelic pathogenic variants in *SLC26A4* (NM_000441.2 c.626G > T (p.Gly209Val), and c.707T > C (p.Leu236Pro)).

DPOAEs were assessed in 19 patients and were present in cases of normal hearing and absent in cases of SNHL or CHL, as expected. For four patients in whom pure-tone data could not be obtained, DPOAEs provided evidence of normal to near-normal hearing sensitivity.

### Middle ear function assessments in patients with GACI

Tympanometry was performed in 19 patients (38 ears, Table 2). Normal tympanograms (type A) were measured in 68.4% of ears (26/38); of these, eight ears had CHL. Type As and type Ad tympanograms were present in 7.9% (n = 3) and 2.6% (n = 1) of ears, respectively. Type As and Ad tympanograms are classic audiological notations which stand for ‘shallow’ (meaning low middle ear compliance) and ‘deep’ (meaning increased middle ear compliance), respectively. Flat (type B) tympanograms were documented in 7.9% (n = 3) of ears and were associated with tympanic membrane perforation (n = 1), patent pressure-equalizing tubes (PET) (n = 1), or middle ear effusion (n = 1). Type C tympanograms suggestive of Eustachian tube dysfunction were recorded in 13.2% (n = 5) of ears.

Acoustic reflexes were assessed in 17 of 38 ears. Of these, the reflex was present at normal levels in 5.9% (n = 1), absent in 76.5% (n = 13), and elevated in 17.7% (n = 3). Six of the ears with absent or abnormal reflexes had normal hearing and normal tympanograms. Five ears with absent reflexes occurred in conjunction with CHL and normal tympanograms.

Of 15 patients with pure-tone threshold data, 10 had sufficient data for OCD categorization; of these, 80% (n = 8) were categorized as having an audiometric pattern consistent with OCD in one or both ears (Table 2). An example of this pattern finding is shown in Fig. 1.

### Newborn hearing screening and hearing aid use in GACI patients

Thirteen patients recalled the results of newborn hearing screening (NBHS): of these, nine patients passed and four did not pass (Table 2). The four who failed the NBHS had HL on NIH examination. Three of the six OCD patients with known results passed the NBHS.

Of the 20 GACI patients, five use hearing aids, four bilaterally and one unilaterally. The median age of initial hearing aid fitting was 9 months (6 months–24 years).

### Otologic findings

Micro-otoscopy revealed abnormal tympanic membranes in 11 of 38 ears examined. Abnormal findings included retracted, thickened, thin/monomeric tympanic membranes and/or presence of serous otitis media (Table 2). Of those with complete audiograms (n = 30 ears), abnormal tympanic membranes were seen in eight ears with HL (seven CHL, one SNHL) and one with normal hearing. The remaining 21 ears with audiometric data had normal tympanic membranes on exam; this included seven with HL (five CHL, two SNHL) and one subclinical conductive loss. Excluding the patient with bilateral EVA, abnormal tympanic membrane findings observed on microscopic otoscopy were significantly correlated with hearing status (p = 0.004, Fisher’s exact test; odds ratio, 22.4; 95% CI, 2.4–261.6).

Approximately two-thirds of patients reported a history of recurrent otitis media (ROM) (13/19, 68.4%). Eleven had information regarding the most recent otitis media episode; of these, nine (81.8%) were in early childhood (< 6yo), and two (18.2%) were in adulthood (> 20 years old). Six individuals (31.6%) had a history of PET placement.

### CT imaging findings in GACI patients

**Fig. 2:**
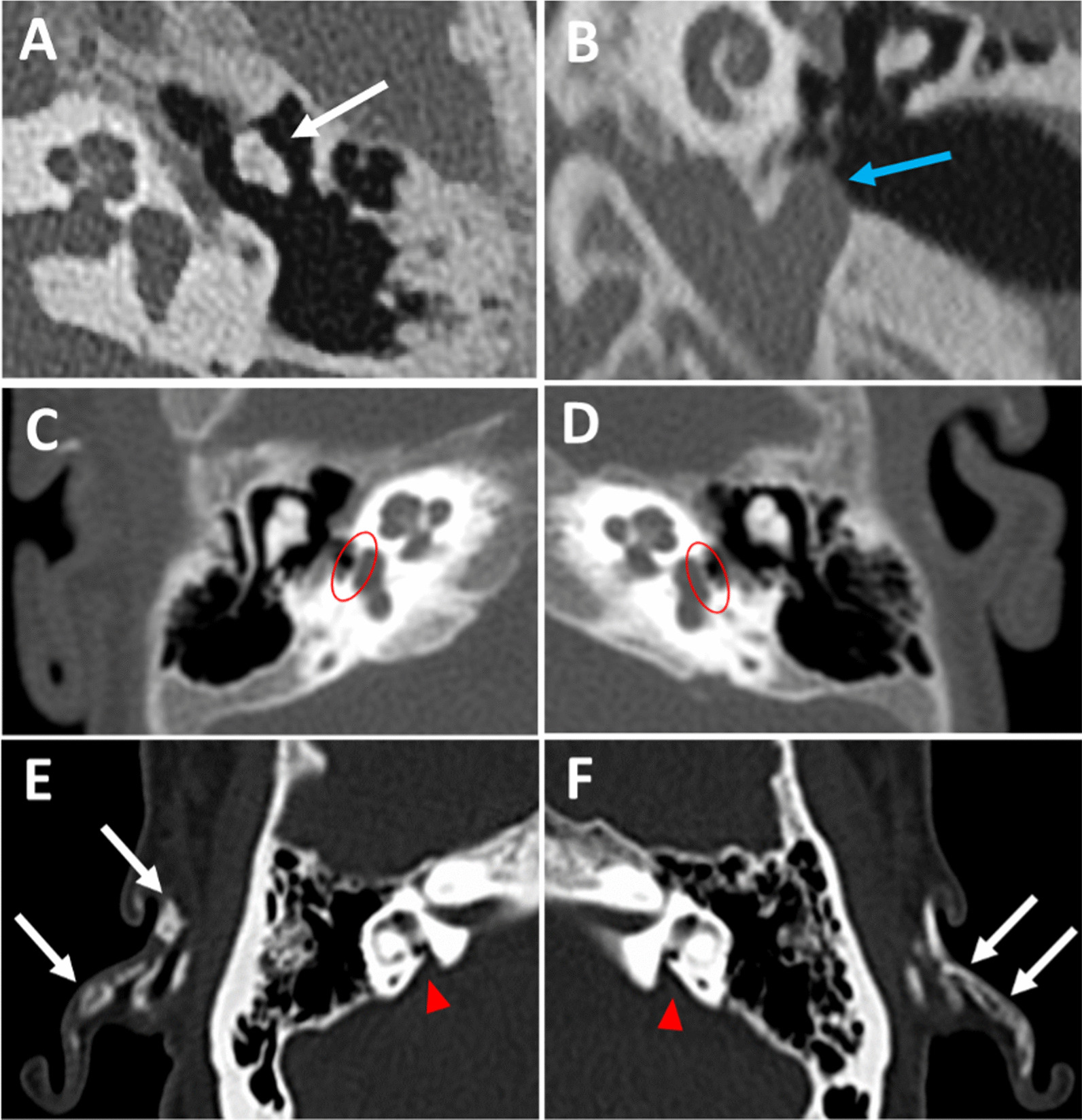
Temporal bone CT images of GACI patients. Abnormal findings (**A**–**D**) in the 25-year-old GACI patient, including an axial view of (**A**) an abnormal malleus and incus in the left ear (white arrow), a coronal view of a (**B**) left-sided high-riding jugular bulb with diverticulum (blue arrow), and axial views of (**C**) right and (**D**) left calcified stapes footplate (encircled). Abnormal findings (**E**–**F**) in the 35-year-old GACI patient were bilateral auricular calcifications (white arrows) and bilateral enlarged vestibular aqueduct (arrowheads)

Head CT was obtained, often for non-otologic reasons, in 11 patients. However, detailed description of the middle ear, ossicles, otic capsule, and inner ear morphology could not be adequately assessed in most patients due to thick section cuts (range 1.0–5 mm). In reviewing the imaging, 54.5% of patients (6/11) or 45.5% of ears (10/22) were observed to have auricular calcification (Fig. 2E–F). No middle ear effusions were identified.

In two patients with bilateral CHL who underwent temporal bone CT scans with adequately thin section cuts, a 25-year-old patient was found to have bilateral auricular calcification, a left-sided abnormal malleus and incus complex (Fig. 2A), left-sided high-riding jugular bulb with diverticulum (Fig. 2B), and bilateral calcified stapes footplates (Fig. 2C–D). Another 37-year-old patient showed bilateral auricular calcification and thickened footplates. Bilateral auricular calcification and bilateral EVA were found in one patient with profound bilateral SNHL (Fig. 2E–F).

### Relationship between hearing loss and other clinical manifestations

There were no significant correlations (Spearman) between biochemical markers, factors related to mineral homeostasis, and measures of cardiovascular burden of disease with the bilateral 4F-PTA. Details can be found in the supplemental materials.

### Quantitative backscattered electron imaging (qBEI) of murine auditory ossicles

**Fig. 3:**
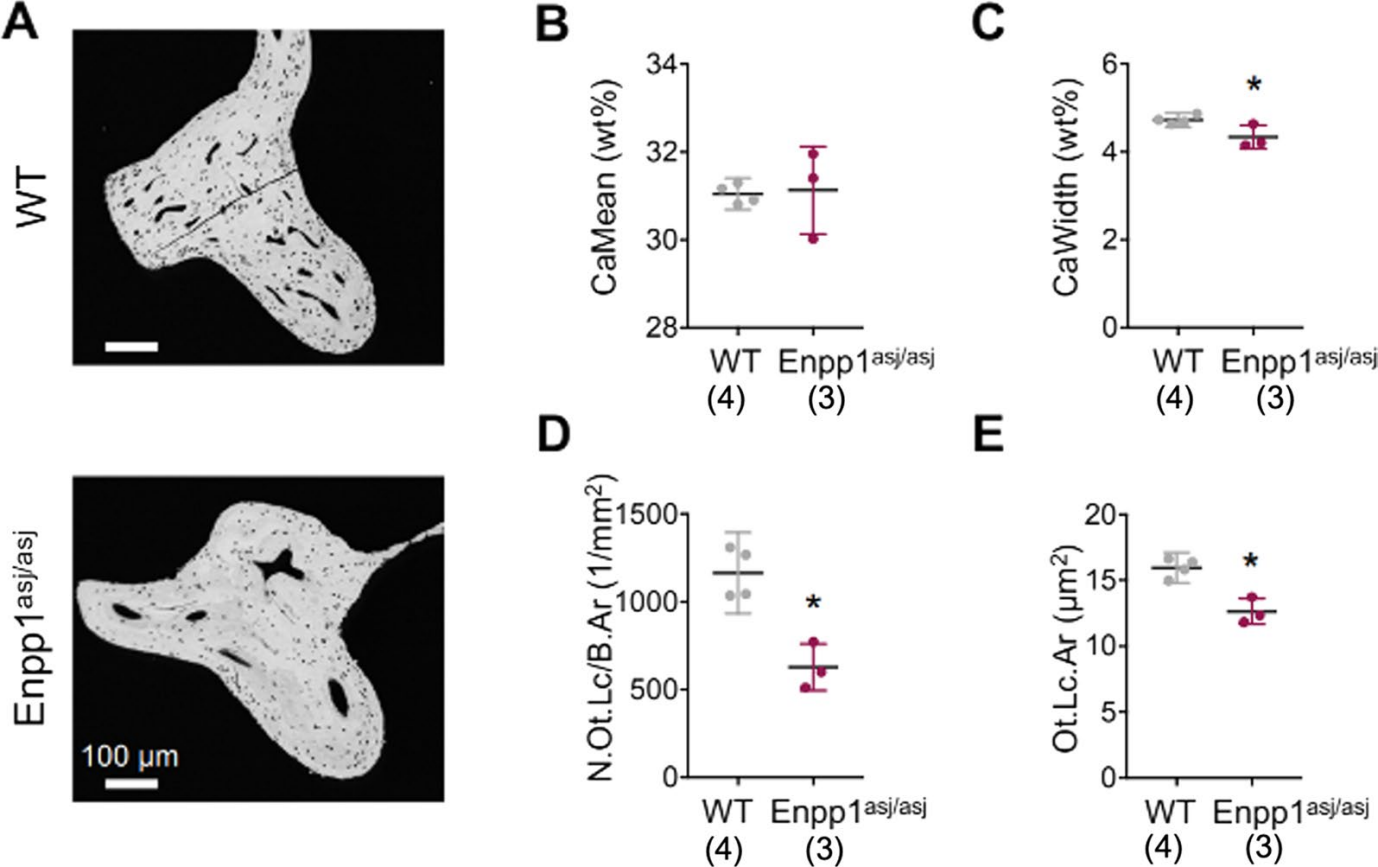
Micromorphology of murine mallei from *Enpp1*^*asj/asj*^ mutant mice. **A** Mallei evaluated by qBEI showed no differences in **B** mean calcium content (or Ca_Mean_; *p* = 0.877) but showed statistically significant decreases in **C** mineralization heterogeneity (or Ca_Width_; *p* = 0.038), **D** number of osteocyte lacunae per bone area (N.Ot.Lc/B.Ar) (*p* = 0.004), and **E** mean osteocyte lacunae area (*p* = 0.004). For the incus and stapes ossicles, please refer to Additional file 1: Figures S1 and S2

Micromorphology, bone mineral density distribution (BMDD), and osteocyte characteristics of murine ossicles were analyzed by qBEI and compared via unpaired student’s *t*-test, between ENPP1^asj/asj^ and WT mice (Fig. 3A). Visual image inspection revealed no major morphologic changes or deformities in the ossicles. In the malleus, no difference was detected in the mean calcium content (Ca_Mean_, 1.13 ± 1.0 vs.31.05 ± 0.22 wt%, p = 0.877; Fig. 3B), while *ENPP1*^*asj/asj*^ mice showed significantly decreased mineralization heterogeneity (Ca_Width_, 4.37 ± 0.27 vs.4.73 ± 1.0 wt%, p = 0.038; Fig. 3C). Analysis of osteocyte characteristics showed a significantly decreased number of osteocyte lacunae per bone area (N.Ot.Lc/B.Ar, 626.8 ± 132.1 vs.1163.0 ± 144.6 /mm2, p = 0.004; Fig. 3D) and decreased mean osteocyte lacunar area (Lc.Ar, 12.63 ± 0.98 vs.15.95 ± 0.73 µm^2^, p = 0.004; Fig. 3E) in ENPP1^asj/asj^ mice compared to WT mice. In the incus and stapes, the Lc.Ar was similarly lower in *ENPP1*^*asj/asj*^ mice than in WT (Additional file 1: Figures S1E & S2E), but the N.Ot.Lc/B.Ar and BMDD parameters showed no significant differences between groups.

## Discussion

This is the first study to systematically characterize hearing impairment in patients with GACI due to ENPP1 deficiency. HL was documented in 53.3% (8/15) of individuals and 50% (15/30) of ears by pure-tone thresholds. CHL occurred in 80% (12/15) and SNHL in 20% (3/15) of HL ears and was mostly mild to moderate in severity. The findings consistent with OCD and recurrent episodes of otitis media were common. In those with head CT imaging, more than half had the presence of auricular calcification.

Of previously reported cases, 15 GACI patients have been noted to have hearing impairment (Additional file 1: Table S3). Of note, the patients in the present study include 10 previously reported by Ferreira and colleagues [2]; discrepancies in these two reports including overlapping patients are likely attributed to the rigorous operational definitions of hearing loss in the current study compared to retrospective extraction from clinical notes in the prior.

The specific cause of HL in ENPP1-deficient GACI remains elusive. However, this population was consistently found to have OCD and high rates of ROM. Three of six patients with OCD passed NBHS, suggesting postnatal development of OCD and CHL, possibly due to abnormal ENPP1-mediated ossicle mineralization. Given the lack of dedicated imaging and surgical exploration, the characterization of OCD remains broad and its cause undetermined. Previously, HL in ENPP1-deficient GACI was hypothesized to result from calcification of arteries supplying the middle and inner ear, ossicular chain fixation, and/or an otitis-mediated calcification of middle ear structures (Additional file 1: Table S3) [2, 4, 7–9]. Our findings indicate that FGF23 levels, hypophosphatemia, age of initiation and duration of rickets treatment, age of initiation and duration of bisphosphonate treatment, number of ongoing and resolved antihypertensive and/or heart failure medications, and age of vascular calcification onset were not correlated with HL.

Micromorphology analyses of murine ossicles support the development of OCD in ENPP1-deficienct GACI, suggesting an early loss of ossicular osteocytes without initiation of bone remodeling. In the present study, comparisons between *ENPP1*^*asj/asj*^ mutant vs. WT murine mallei demonstrated significant decreases in the Ca_Width_, number of osteocyte lacunae per bone area, and osteocyte lacunae area, with similar trends in the incus. The decreased Ca_Width_ translates to decreased heterogeneity in bone matrix mineralization and contrasts with the expectation of greater variation as typically seen in osteomalacia due to increased osteoid deposition [16]. The decreased number of osteocyte lacunae per bone area and lacunae area are consistent with early ossicular aging and have been associated with osteocyte apoptosis in prior studies [17]. It is known that ENPP1-deficient mice have fewer and smaller osteocyte lacunae [18]. In mice, hypophosphatemia has been shown to regulate perilacunar remodeling, and its impairment is associated with HL [19, 20]. Additionally, HL in GACI was previously studied in the *ENPP1*^*asj/asj*^ mice, the main findings being otitis media and pathologic calcification in the middle ear, including over-ossification of the round window niche, fusion of the malleus and incus, tympanosclerosis, and a thickened and over-calcified stapedial artery [9]. Taken together, these results suggest an early loss of ossicular osteocytes with loss of perilacunar bone remodeling, leading to ossicular chain dysfunction and conductive hearing loss.

Ossicular deterioration in CHL has been demonstrated in other mouse models of FGF23-mediated hypophosphatemia, such as those caused by involvement of Dmp1, a regulator of phosphate homeostasis and mineralization [21]. However, HL in patients with X-linked hypophosphatemia (XLH) is often asymmetric and sensorineural rather than conductive, although some present with MHL. [22, 23]. Morphologically, XLH patients have generalized osteosclerosis and significant thickening of the petrous temporal bone [22, 23]. Prevalence of HL in XLH ranges from 14 to 76% [23–27]. In contrast, there were only two patients in our cohort with SNHL, one of whom had concurrent bilateral EVA due to variants in *SLC26A4*. Given the predominance of CHL in this cohort, a second etiology may thus be possible in the setting of SNHL.

There is a high prevalence of ROM (56.5%) and increased frequency of PET placement (26.1%) in GACI vs. the general population (8.9%), highlighting clinically significant ROM necessitating intervention [28]. These findings support previous observations in the *ENPP1*^*asj/asj*^ mouse, where otitis media with effusion was observed in conjunction with progressive HL [9]. All mutant mice exhibited thickened middle ear epithelium with fibrous polyps and increased mucin-secreting goblet cell [9]. ROM may predispose patients to abnormal tympanic membranes, scar tissue in the middle ear space, or ossicular chain fixation/erosion leading to chronic HL [29, 30].

Auricular calcification was observed in more than half of patients with head imaging, the most common CT finding in our cohort and previously observed in murine models [31].

Strengths of this study include cohort size given disease rarity and the consistent and comprehensive audiologic and otologic evaluations performed at a single institution. Limitations include those inherent to retrospective studies of rare diseases, the cross-sectional design, and inadequate power for certain statistical analyses. It is thus possible that certain analyses for which we found no association could have reached statistical significance with a larger cohort of patients. Future animal studies could establish whether the administration of recombinant enzyme replacement therapy, shown to prevent the cardiovascular and skeletal complications of the disease, could prevent or improve hearing impairment [28, 29].

## Conclusions

Hearing impairment is frequent in patients with ENPP1-deficient GACI, ranging from mild to moderate CHL, and less commonly, SNHL. Novel findings of high rates of suspected OCD and ROM are consistent with a GACI murine model and suggest that patients with ENPP1-deficient GACI are at increased risk for progressive HL, warranting ongoing formal audiologic and otolaryngologic assessments. Early detection of HL may lead to timely treatment, minimizing speech and cognitive sequelae, especially in young patients. While HL from ROM could be treated with PET placement, HL from OCD may be managed with hearing aids and/or assistive listening devices. Select cases may benefit from surgical intervention to restore CHL, including ossicular chain reconstruction and bone conduction implantation. This study highlights the importance of early comprehensive audiologic evaluation with continued monitoring in individuals with GACI.

## Supplementary Information

**Additional file 1: Table S1**. Audiology interpretation criteria. 4F-PTA, four frequency (.5, 1, 2, 4 kHz) pure tone average; 3F-PTA, three frequency (.5, 1, 2kHz) pure tone average.

## Data Availability

There is no data presented in this study that requires submission to a public repository.

## Abbreviations

GACI: Generalized Arterial Calcification of Infancy
ENPP1: Ectonucleotide pyrophosphatase/phosphodiesterase-1
PP_i_: Pyrophosphate
HL: Hearing loss
CHL: Conductive hearing loss
SNHL: Sensorineural hearing loss
MHL: Mixed hearing loss
4F-PTA: 4-Frequency pure-tone average
3F-PTA: 3-Frequency pure-tone average
DPOAE: Distortion product otoacoustic emissions
OCD: Ossicular chain dysfunction
PET: Pressure-equalizing tympanostomy tubes

## References

[1] Rutsch F, Böyer P, Nitschke Y (2008). Hypophosphatemia, hyperphosphaturia, and bisphosphonate treatment are associated with survival beyond infancy in Generalized Arterial Calcification of Infancy. Circ Cardiovasc Genet.

[2] Ferreira CR, Hackbarth ME, Ziegler SG (2021). Prospective phenotyping of long-term survivors of Generalized Arterial Calcification of Infancy (GACI). Genet Med.

[3] Chong CR, Hutchins GM (2008). Idiopathic infantile arterial calcification: the spectrum of clinical presentations. Pediatr Dev Pathol.

[4] Nitschke Y, Baujat G, Botschen U (2012). Generalized Arterial Calcification of Infancy and pseudoxanthoma elasticum can be caused by mutations in either ENPP1 or ABCC6. Am J Hum Genet.

[5] Gopalakrishnan S, Shah S, Apuya JS, Martin T (2008). Anesthetic management of a patient with idiopathic arterial calcification of infancy and fused cervical spine. Paediatr Anaesth.

[6] Le Boulanger G, Labrèze C, Croué A (2010). An unusual severe vascular case of pseudoxanthoma elasticum presenting as Generalized Arterial Calcification of Infancy. Am J Med Genet A.

[7] Brachet C, Mansbach AL, Clerckx A, Deltenre P, Heinrichs C (2014). Hearing loss is part of the clinical picture of ENPP1 loss of function mutation. Horm Res Paediatr.

[8] Lorenz-Depiereux B, Schnabel D, Tiosano D, Häusler G, Strom TM (2010). Loss-of-function ENPP1 mutations cause both Generalized Arterial Calcification of Infancy and autosomal-recessive hypophosphatemic rickets. Am J Hum Genet.

[9] Tian C, Harris BS, Johnson KR (2016). Ectopic mineralization and conductive hearing loss in Enpp1asj mutant mice, a new model for otitis media and tympanosclerosis. PLoS ONE.

[10] Mazzoli MG, Van Camp GU, Newton V, Giarbini N, Declau F, Parving A (2003). Recommendations for the description of genetic and audiological data for families with nonsyndromic hereditary hearing impairment. Audiolog Med..

[11] Carhart R, Ventry IM, Chaiklin JB, Dixon RF (1971). Effects of stapes fixation on bone-conduction response. Hearing measurement: a book of readings.

[12] Li H, Durbin R (2009). Fast and accurate short read alignment with Burrows–Wheeler transform. Bioinformatics.

[13] Van de Auwera G, O’Connor BD (2020). Genomics in the cloud: using Docker, GATK, and WDL in Terra.

[14] Cingolani P, Platts A, le Wang L (2012). A program for annotating and predicting the effects of single nucleotide polymorphisms, SnpEff: SNPs in the genome of *Drosophila melanogaster* strain w1118; iso-2; iso-3. Fly (Austin).

[15] Li Q, Guo H, Chou DW, Berndt A, Sundberg JP, Uitto J (2013). Mutant Enpp1asj mice as a model for Generalized Arterial Calcification of Infancy. Dis Model Mech.

[16] Roschger P, Lombardi A, Misof BM (2010). Mineralization density distribution of postmenopausal osteoporotic bone is restored to normal after long-term alendronate treatment: qBEI and sSAXS data from the fracture intervention trial long-term extension (FLEX). J Bone Miner Res.

[17] Rolvien T, Schmidt FN, Milovanovic P (2018). Early bone tissue aging in human auditory ossicles is accompanied by excessive hypermineralization, osteocyte death and micropetrosis. Sci Rep.

[18] Hajjawi MO, MacRae VE, Huesa C (2014). Mineralisation of collagen rich soft tissues and osteocyte lacunae in Enpp1(−/−) mice. Bone.

[19] Akil O, Hall-Glenn F, Chang J (2014). Disrupted bone remodeling leads to cochlear overgrowth and hearing loss in a mouse model of fibrous dysplasia. PLoS ONE.

[20] Tokarz D, Martins JS, Petit ET, Lin CP, Demay MB, Liu ES (2018). Hormonal regulation of osteocyte perilacunar and canalicular remodeling in the Hyp mouse model of X-linked hypophosphatemia. J Bone Miner Res.

[21] Lv K, Huang H, Yi X (2017). A novel auditory ossicles membrane and the development of conductive hearing loss in Dmp1-null mice. Bone.

[22] O'Malley SP, Adams JE, Davies M, Ramsden RT (1988). The petrous temporal bone and deafness in X-linked hypophosphataemic osteomalacia. Clin Radiol.

[23] Chesher D, Oddy M, Darbar U (2018). Outcome of adult patients with X-linked hypophosphatemia caused by PHEX gene mutations. J Inherit Metab Dis.

[24] Boneh A, Reade TM, Scriver CR, Rishikof E (1987). Audiometric evidence for two forms of X-linked hypophosphatemia in humans, apparent counterparts of Hyp and Gy mutations in mouse. Am J Med Genet.

[25] Meister M, Johnson A, Popelka GR, Kim GS, Whyte MP (1986). Audiologic findings in young patients with hypophosphatemic bone disease. Ann Otol Rhinol Laryngol.

[26] Davies M, Kane R, Valentine J (1984). Impaired hearing in X-linked hypophosphataemic (vitamin-D-resistant) osteomalacia. Ann Intern Med.

[27] Weir N (1977). Sensorineural deafness associated with recessive hypophosphataemic rickets. J Laryngol Otol.

[28] Simon AE, Boss EF, Zelaya CE, Hoffman HJ (2017). Racial and ethnic differences in receipt of pressure equalization tubes among US children, 2014. Acad Pediatr.

[29] Kurihara A, Toshima M, Yuasa R, Takasaka T (1991). Bone destruction mechanisms in chronic otitis media with cholesteatoma: specific production by cholesteatoma tissue in culture of bone-resorbing activity attributable to interleukin-1 alpha. Ann Otol Rhinol Laryngol.

[30] Chole RA, McGinn MD, Tinling SP (1985). Pressure-induced bone resorption in the middle ear. Ann Otol Rhinol Laryngol.

[31] Khan T, Sinkevicius KW, Vong S (2018). ENPP1 enzyme replacement therapy improves blood pressure and cardiovascular function in a mouse model of Generalized Arterial Calcification of Infancy. Dis Model Mech.

