# Supplementary material for: Characterization of hearing-impairment in Generalized Arterial Calcification of Infancy (GACI)
Source: Orphanet J Rare Dis. 2022 Jul 19;17:273. doi: 10.1186/s13023-022-02410-w (PMC9295326; doi:10.1186/s13023-022-02410-w)
Supplement: Supplementary file 1 — Additional file 1: Table S1. Audiology interpretation criteria. 4F-PTA, four frequency (.5, 1, 2, 4 kHz) pure tone average; 3F-PTA, three frequency (.5, 1, 2kHz) pure tone average. [file 13023_2022_2410_MOESM1_ESM.docx]

**SUPPLEMENTAL METHODS**

**Tympanometry and acoustic stapedial reflexes**

Tympanometry and acoustic stapedial reflex thresholds (0.5, 1, and 2 kHz), were obtained using a TympStar immittance bridge (Grason Stadler, Inc.). Data were interpreted using published reference ranges^1,2^ and tympanogram types (A, As, Ad, B, C) were assigned based on peak admittance and peak pressure (Table S1).

**Distortion product otoacoustic emissions (DPOAE)**

DPOAE were evaluated using an Echoport Otoacoustics Emission System (ILO v6, Otodynamics, Inc.). Eliciting stimuli were presented in quarter octave bands from 842 to 7996 Hz, using an f2/f1 ratio of 1.2 and L1 and L2 of 65- and 55-dB SPL, respectively. DPOAEs were interpreted as present when the signal-to-noise ratio exceeded 6 dB.

**Murine model**

The animals were housed in pathogen-free conditions and fed a regular chow diet with access to food and water *ad libitum*. Animal care and maintenance were provided through Yale University Animal Resource Center (YARC) at Yale University (New Haven), and all procedures were approved by the Animal Care and Use Committee of Yale University and complied with the US NIH guide for the care and use of laboratory animals. Ossicles were extracted at 23 weeks of age under a stereomicroscope, dehydrated in an ascending ethanol series and embedded without de-calcification in methyl-methacrylate. Quantitative backscattered electron imaging (qBEI) was performed using a scanning electron microscope (LEO 435 VP, LEO Electron Microscopy Ltd.; Cambridge, England) with a backscattered electron detector (Type 202; K.E. Developments Ltd.; Cambridge, UK) as described previously^3,4^. Briefly, the working distance of the scanning microscope was operated constantly at 20 kV and 680 pA. Analysis of qBEI images was performed by using ImageJ analysis software (ImageJ 1.42, NIH, Bethesda, USA) and a customized MATLAB-based program (TheMathWorks, Inc., Natick, USA). Based on the gray values, which are proportional to the calcium content, the mean calcium content (Ca_Mean_, wt%) and the mineralization heterogeneity (Ca_Width_, wt%) were determined. Furthermore, the number of the osteocyte lacunae (N.Ot.Lc/B.Ar, 1/mm2) and mean osteocyte lacunar area (Lc.Ar, µm^2^) were measured using ImageJ. Statistical analysis was performed using unpaired student’s *t*-test.

**SUPPLEMENTAL RESULTS**

**Relationship between hearing and factors related to GACI status**

For purposes of Spearman correlation, the patient with EVA and pathogenic variants in *SLC26A4* was excluded from analysis**.**

**FGF23**

Intact FGF23 (iFGF23) was measured in 91.3% (21/23) of all GACI patients and 75% (6/8) of those with hearing impairment. The median iFGF23 value in all patients was 75 pg/mL (range 13-168) and the median iFGF23 in HL patients was 83.5 pg/mL (range 29-148); normal values for iFGF23 are typically between 22-63 pg/mL. Thirteen patients had intact FGF23 results and complete audiograms. One patient was excluded from analysis due to bilateral EVA and pathogenic variants in *SLC26A4*. Spearman correlation did not reveal any significant association between intact FGF23 and average of binaural AC 4F-PTA (p=0.46).

C-terminal FGF23 (cFGF23) was measured in 73.9% (17/23) of all patients and 87.5% (7/8) of those with HL. Excluding one patient with extremely elevated cFGF23 due to prior burosumab use, the median cFGF23 was 167 RU/mL (range 73-426) and the median in HL patients was 176 (range 138 to 143); normal values for cFGF23 are below or equal to 230 RU/mL in children and <=180 RU/mL in adults. Eleven patients had cFGF23 results and complete audiograms. Excluding one patient with prior burosumab use and the patient with bilateral EVA, Spearman correlation between cFGF23 levels and average of binaural AC 4F-PTA was not significant (p=0.35).

**Hypophosphatemia**

60.9% (14/23) of all GACI patients were diagnosed with hypophosphatemia while 75% (6/8) of HL patients were diagnosed with hypophosphatemia. Plasma phosphate levels obtained at the time of audiology visit were normalized to Z-scores using the provided normal range. The median phosphate Z-score in all 23 patients was -2.3 (-5.2 to 1.4). The median phosphate Z-score in HL individuals was -2.4 (-5.2 to -0.8). Spearman correlation between phosphate Z-scores and average of binaural AC 4F-PTA was not significant (p=0.75).

**Treatment of Hypophosphatemic Rickets**

Rickets treatment with calcitriol and phosphorus supplementation was administered in 56.2% (13/23) of all patients and 75% (6/8) in all HL patients. Of those receiving rickets treatment, the median age of initiation was 6.7 years in all patients and 6.3 years in all HL patients. Twelve patients had records of rickets treatment and complete audiograms; Spearman correlation between age of rickets treatment initiation and binaural AC 4F-PTA was not significant (p=0.35).

In patients receiving treatment for rickets, the median duration of treatment, from initiation to time of most recent audiologic evaluation, was 1.6 years (range 0.13-29.7). Spearman correlation between duration of rickets treatment and average of binaural AC 4F-PTA was not significant (p=0.26).

**Cardiovascular burden of disease**

Age of onset of calcification

Thirteen patients had information about age of calcification onset and complete audiology evaluations. Excluding the EVA patient, Spearman correlation between age of calcification onset and average of binaural AC 4F-PTA was not significant (p=0.11).

Number of ongoing and resolved antihypertensive and/or heart failure medications

Fifteen patients had medication information and complete audiology evaluations. Spearman correlation between number of ongoing and resolved antihypertensive and/or heart failure medications and average of binaural AC 4F-PTA was not significant (p=0.53, p=0.98 respectively).

**Bisphosphonate (BP) use**

The prevalence of BP use is 60.9% (14/23) in all GACI patients and 50% (4/8) in all HL patients. Of those receiving BPs, the median age of initiation was 20 days in all patients and 25 days in HL patients; the mean duration of BP use is 16.4 months in all patients, and 12.5 months in HL patients. Administered BPs included etidronate (10 patients), pamidronate (8 patients), and risedronate (1 patient); some patients received more than one type of bisphosphonate during their lifetimes. Eight patients had records of BP initiation and complete audiograms. Spearman correlation between age of BP initiation and duration of use and average of binaural 4F-PTAs was not significant (p=0.55 and p=0.94, respectively).

**Supplemental Tables**

**Table S1. Audiology interpretation criteria^1,5^**

| **Degree of Hearing Loss** | | |
| --- | --- | --- |
| ***Descriptor*** | ***Criteria^5^*** | |
| Mild | 4F-PTA >20 and ≤40 dBHL | |
| Moderate | 4F-PTA >40 and ≤70 dBHL | |
| Severe | 4F-PTA >70 and ≤90 dBHL | |
| Profound | 4F-PTA >90 dBHL | |
| **Type of Hearing Loss** | | |
| ***Descriptor*** | ***Criteria^5^*** | |
| Conductive | 3F-PTA: air-bone gap ≥10 dB and bone conduction ≤20 dBHL | |
| Sensorineural | 3F-PTA: air-bone gap <10 dB and bone conduction >20 dBHL | |
| Mixed | 3F-PTA: air-bone gap ≥15 dB with bone conduction >20 dBHL | |
| **Tympanogram types** | | |
| ***Descriptor (tympanogram type)*** | ***Criteria^1^*** | |
|  | ***Child (3-5 Years)*** | ***Adult*** |
| Normal (type A) | 0.2-0.9 cc, > -100 daPa | 0.3-1.4 cc, > -100 daPa |
| Hypermobile (type Ad) | > 0.9 cc, > -100 daPa | >1.4 cc, > -100 daPa |
| Hypomobile (type As) | < 0.2 cc, > -100 daPa | < 0.3 cc, > -100 daPa |
| Flat (type B) | No mobility, no peak | >1.4 cc, > -100 daPa |
| Negative pressure (type C) | < -100 daPa | < -100 daPa |

4F-PTA, four frequency (.5, 1, 2, 4 kHz) pure tone average; 3F-PTA, three frequency (.5, 1, 2 kHz) pure tone average

**Table S2. Molecular description of GACI patients**

| Age (yrs) | **Sex** | **HL phenotype** | | **Molecular Description** | | | |
| --- | --- | --- | --- | --- | --- | --- | --- |
|  |  | RE | LE | **Gene** | **Mutation(s)** | **Protein(s)** | **Domain(s)** |
| 0.7 | M | . | . | *ENPP1* | c.913C>A | p.(Pro305Thr) | PDE |
|  |  |  |  | *ENPP1* | c.2662C>T | p.(Arg888Trp) | Nuclease-like |
| 2.0 | F | . | . | *ENPP1* | c.1652A>G | p.(Tyr551Cys) | PDE |
|  |  |  |  | *ENPP1* | c.1737G>C | p.(Leu579Phe) | PDE |
| ➢4.1 | F | CHL | CHL | *ENPP1* | c.2735T>C | p.(Leu912Ser) | Nuclease-like |
|  |  |  |  | *ENPP1* | 3.4 kb deletion of exon 6 | NA | PDE |
| ➢4.2 | M | . | . | *ENPP1* | c.1441C>T | p.(Arg481Trp) | PDE |
|  |  |  |  | *ENPP1* | c.2713_2717del | p.(Lys905Alafs*16) | Nuclease-like |
| 4.5 | F | NH | NH | *ENPP1* | c.1438T>C | p.(Cys480Arg) | PDE |
|  |  |  |  | *ENPP1* | c.2414G>T | p.(Gly805Val) | Nuclease-like |
| ➢5.1 | M | NH | CHL | *ENPP1* | c.2320C>T | p.(Arg774Cys) | Nuclease-like |
|  |  |  |  | *ENPP1* | c.2662C>T | p.(Arg888Trp) | Nuclease-like |
| 5.8 | F | NH | NA | *ENPP1* | c.1652A>G | p.(Tyr551Cys) | PDE |
|  |  |  |  | *ENPP1* | c.2330A>G | p.(His777Arg) | Nuclease-like |
| ➢6.3 | F | NH | NH | *ENPP1* | c.1438T>C | p.(Cys480Arg) | PDE |
|  |  |  |  | *ENPP1* | c.2414G>T | p.(Gly805Val) | Nuclease-like |
| ➢7.0† | M | CHL* | CHL* | *ENPP1* | c.1441C>T | p.(Arg481Trp) | PDE |
|  |  |  |  | *ENPP1* | c.2312-5_2313del | NA | NA |
| ➢7.9† | F | CHL* | CHL* | *ENPP1* | c.783C>G | (p.Tyr261*) | PDE |
|  |  |  |  | *ENPP1* | c.1756G>A | (p.Gly586Arg) | PDE |
| ➢8.2 | M | SubCl | NH | *ABCC6* | c.3940C>T | p.(Arg1314Trp) | na |
|  |  |  |  | *ABCC6* | c.3940C>T | p.(Arg1314Trp) | na |
| ➢8.9 | F | SNHL | CHL | *ENPP1* | c.1538A>G | p.(Tyr513Cys) | PDE |
|  |  |  |  | *ENPP1* | c.1538A>G | p.(Tyr513Cys) | PDE |
| 9.0 | M | . | . | *ENPP1* | c.913C>A | p.(Pro305Thr) | PDE |
|  |  |  |  | *ENPP1* | c.1499A>C | p.(His500Pro) | PDE |
| 9.7† | M | CHL* | CHL* | *ABCC6* | c.3940C>T | p.(Arg1314Trp) | na |
|  |  |  |  | *ABCC6* | c.3940C>T | p.(Arg1314Trp) | na |
| 13.1 | F | NH | NH | *ENPP1* | c.1441C>T | p.(Arg481Trp) | PDE |
|  |  |  |  | *ENPP1* | c.2312-5_2313del | NA | Nuclease-like |
| ➢25.5† | M | CHL | CHL* | *ENPP1* | c.1412A>G | p.(Tyr471Cys) | PDE |
|  |  |  |  | *ENPP1* | c.1442G>A | p.(Arg481Gln) | PDE |
| 26.3 | F | NH | NHL | *ENPP1* | c.749C>T | p.(Pro250Leu) | PDE |
|  |  |  |  | *ENPP1* | c.913C>A | p.(Pro305Thr) | PDE |
| 35.7†^α^ | F | SNHL* | SNHL* | *ENPP1* | c.715+1G>C | NA | PDE |
|  |  |  |  | *ENPP1* | c.2376T>A | (p.Asn792Lys) | PDE |
| 38.5† | F | NH | NA | *ENPP1* | c.715+1G>C | NA | PDE |
|  |  |  |  | *ENPP1* | c.2376T>A | (p.Asn792Lys) | Nuclease-like |

CHL, conductive hearing loss; HL, hearing loss; LE, left ear; NA, not applicable; NH, normal hearing; NR, not reported; RE, right ear; SNHL, sensorineural hearing loss; SubCl, subclinical air-bone gaps

➢ Arrows denote nine of ten patients were previously reported in Ferreira et al., 2021. The 10^th^ previously reported patient was removed from this study due to cerumen impaction.

**†**Daggers denote hearing aid use in the patient

*Asterisks denote hearing aid use in the specific ear

**^α^**This 35.7-year-old female patient has bilateral EVA and biallelic pathogenic variants in *SLC26A4.*

Periods represent insufficient data.

**Table S3. Summary of previously described HL in GACI**

| Study | Type | Type of HL | Details of study | Cause/Hypothesis for HL |
| --- | --- | --- | --- | --- |
| Lorenz-Depiereux et al., 2010 | Case report | SNHL | 4-year-old boy | SNHL hypothesized due to calcification of the arteries supplying the inner ear |
| Nitschke et al., 2012 | Case series (n=3) | CHL | 9-year-old boy | Stapedovestibular ankylosis |
|  |  | CHL | 5-year-old boy | Stapedovestibular ankylosis |
|  |  | Unspecified, progressive | 9-year-old boy | None provided |
| Brachet et al., 2014 | Case report | MHL with eventual improvement in CHL but persistence of SNHL | HL diagnosed at 9 days of life. PET tubes at age 1.9 and 3.7 years. Hearing aids at age 4.  CT scan at age 25 did not identify middle ear calcification, only external ear cartilage calcification. | CHL hypothesized to be due to middle ear calcification  MHL cause unknown; SNHL component hypothesized to occlusion of cochlear vasculature |
| Ferreira et al., 2021 | Descriptive prospective phenotyping study | *10 patients with HL  7 CHL  3 MHL | Median age of diagnosis at 3.7 in all HL individuals.  Kaplan-Meier estimate of developing hearing loss of 20% by age two years, 50% by four years, and 75% over a lifetime | None provided |

*These 10 patients were included in the evaluation of the present study.

**SUPPLEMENTAL FIGURES**


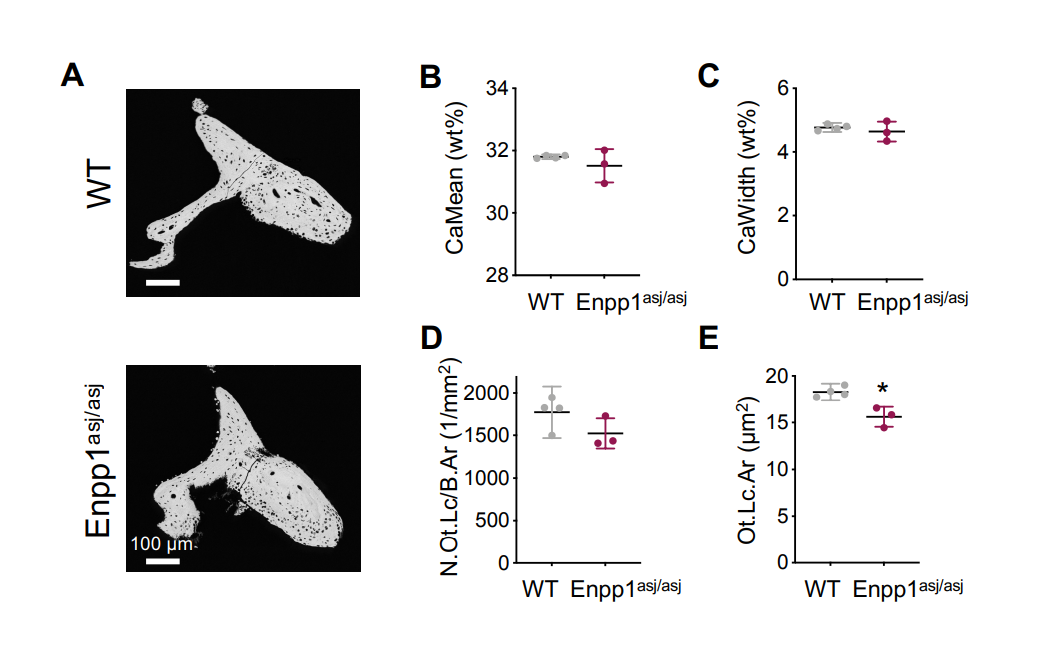


**Figure S1. Micromorphology of murine incus from *ENPP1^asj/asj^* mutant mice**. qBEI analysis show only statistically significant decreases in [E] mean osteocyte lacunae area (p = 0.0075).


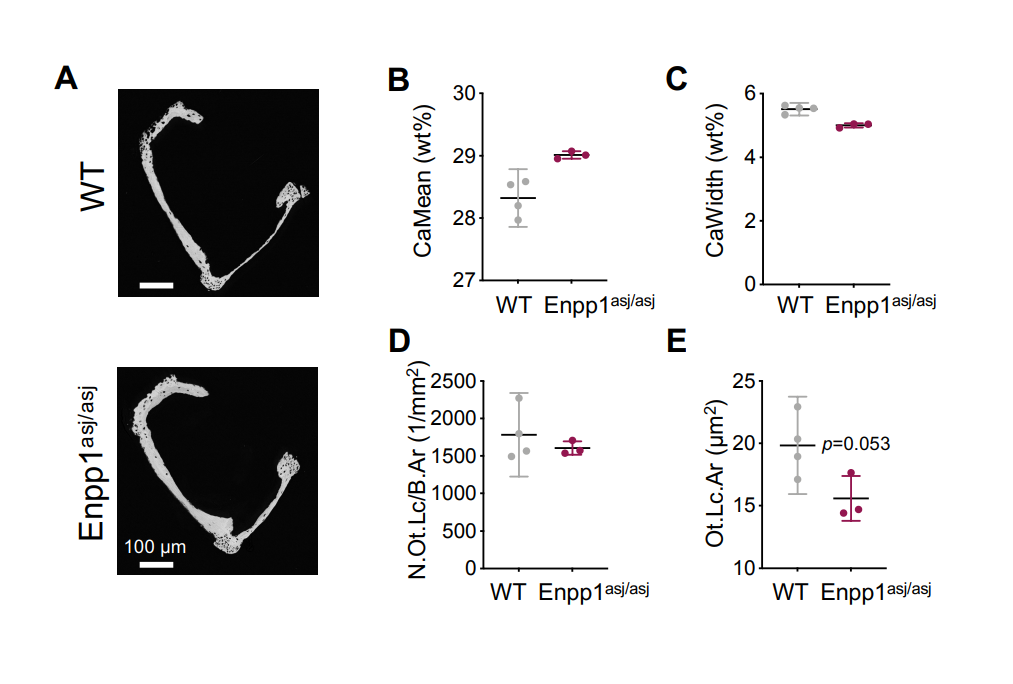


**Figure S2. Micromorphology of murine stapes from *ENPP1^asj/asj^* mutant mice**.

qBEI analysis did not demonstrate statistically significant differences in the stapes.

**Supplemental References**

1. Margolis RH, Heller JW. Screening tympanometry: criteria for medical referral. *Audiology*. 1987;26(4):197-208. doi:10.3109/00206098709081549
2. Gelfand SA, Schwander T, Silman S. Acoustic reflex thresholds in normal and cochlear-impaired ears: effects of no-response rates on 90th percentiles in a large sample. *J Speech Hear Disord*. May; 55(2):198-205. doi: 10.1044/jshd.5502.198. PMID: 2329784.
3. Koehne T, Vettorazzi E, Kusters N, Luneburg R, Kahl-Nieke B, Puschel K, Amling M, Busse B. Trends in trabecular architecture and bone mineral density distribution in 152 individuals aged 30-90 years. Bone. 2014;66:31-8.
4. Rolvien T, Schmidt FN, Milovanovic P, et al. Early bone tissue aging in human auditory ossicles is accompanied by excessive hypermineralization, osteocyte death and micropetrosis. *Sci Rep*. Jan 30 2018;8(1):1920. doi:10.1038/s41598-018-19803-2
5. Mazzoli M, van Camp G, Newton V, Giarbini N, Declau F, Parving A. Recommendations for the Description of Genetic and Audiological Data for Families with Nonsyndromic Hereditary Hearing Impairment. *Audiological Medicine*. 07/11 2009;1:148-150.
